# Electrospun Photodynamic Antibacterial Konjac Glucomannan/Polyvinylpyrrolidone Nanofibers Incorporated with Lignin-Zinc Oxide Nanoparticles and Curcumin for Food Packaging

**DOI:** 10.3390/foods13132007

**Published:** 2024-06-25

**Authors:** Huimin Xiao, Lin Wang, Nitong Bu, Jie Duan, Jie Pang

**Affiliations:** 1 College of Food Science, Fujian Agriculture and Forestry University, Fuzhou 350002, China; 2 Key Laboratory of Colloid and Interface Chemistry, Ministry of Education, Shandong University, Jinan 250100, China; 3 State Key Laboratory of Chemical Resource Engineering, Beijing Advanced Innovation Center for Soft Matter Science and Engineering, Beijing University of Chemical Technology, Beijing 100029, China

**Keywords:** konjac glucomannan, polyvinylpyrrolidone, composite nanoparticles, electrospun nanofibers, photodynamic antibacterial, food packaging

## Abstract

Due to the growing concerns surrounding microbial contamination and food safety, there has been a surge of interest in fabricating novel food packaging with highly efficient antibacterial activity. Herein, we describe novel photodynamic antibacterial konjac glucomannan (KGM)/polyvinylpyrrolidone (PVP) nanofibers incorporated with lignin-zinc oxide composite nanoparticles (L-ZnONPs) and curcumin (Cur) via electrospinning technology. The resulting KGM/PVP/Cur/L-ZnONPs nanofibers exhibited favorable hydrophobic properties (water contact angle: 118.1°), thermal stability, and flexibility (elongation at break: 241.9%). Notably, the inclusion of L-ZnONPs and Cur endowed the nanofibers with remarkable antioxidant (ABTS radical scavenging activity: 98.1%) and photodynamic antimicrobial properties, demonstrating enhanced inhibitory effect against both *Staphylococcus aureus* (inhibition: 12.4 mm) and *Escherichia coli* (12.1 mm). As a proof-of-concept study, we evaluated the feasibility of applying nanofibers to fresh strawberries, and the findings demonstrated that our nanofibers could delay strawberry spoilage and inhibit microbial growth. This photodynamic antimicrobial approach holds promise for design of highly efficient antibacterial food packaging, thereby contributing to enhanced food safety and quality assurance.

## 1. Introduction

Food spoilage caused by microorganisms leads to significant food wastage and substantial economic losses [1]. Therefore, it is imperative to explore antimicrobial preservation methods for food to effectively control spoilage and deterioration [2]. One such method involves the application of antibacterial agents directly onto the food surface [3]. However, this approach entails potential concerns as it necessitates the contact of food with the antimicrobial agents, which can result in the generation of toxic substances and compromise food quality [4]. Nowadays, there has been a growing interest in the development of food packaging, which offers a promising solution to mitigate the potential toxicity while ensuring food quality [5]. Nevertheless, the antimicrobial performance of this packaging still requires improvement. Therefore, it is imperative to implement measures that enhance its antimicrobial capabilities, effectively inhibiting the growth of microorganisms and extending the shelf life of food products [6].

Photodynamic inactivation (PDI) represents an innovative antimicrobial approach that can be seamlessly integrated with packaging to enhance its antimicrobial properties [7]. A key advantage of PDI is its ability to overcome bacterial drug resistance, a critical concern in traditional antimicrobial methods [8,9]. Nano-zinc oxide (Nano-ZnO) exhibits a high specific surface area, photocatalytic capabilities, and robust antibacterial activity [10,11]. Furthermore, it has been identified as a potential photosensitizer, capable of producing reactive oxygen species (ROS) when exposed to light at specific wavelengths [12]. Presently, it finds applications in diverse fields such as food packaging [13], sensors [14], batteries [15], and other applications. However, its utility is hindered by drawbacks like low stability and challenging dispersion [16,17]. Lignin is the second most abundant plant constituent and contains a variety of functional groups, including hydroxyl, carboxyl, and carbonyl groups [18,19]. Previous research has shown that lignin can improve the dispersion of Nano-ZnO [20]. Additionally, by combining Nano-ZnO with lignin for nanoparticle preparation, a synergistic effect can be achieved to augment antimicrobial activity [21]. Curcumin (Cur), a naturally occurring fat-soluble polyphenolic compound extracted from turmeric, possesses multiple beneficial physiological functions [22,23]. Furthermore, curcumin exhibits hydrophobic, antioxidant, and antimicrobial properties which have the potential to further enhance the functional properties of the films, thereby expanding their applications [24]. It is worth mentioning that the addition of these natural extracts has an effect on the water vapor permeability (WVP) of nanofibers; this effect is not dependent on the hydrophobicity of the natural extracts themselves; rather, it is also related to the nanofiber substrate and interactions [25]. For example, hydrophobic curcumin increases the WVP of cellulose/chitin nanofibers, whereas water-soluble anthocyanins decrease the WVP of pullulan/polyvinyl alcohol nanofibers [26,27].

So far, electrospun technology has become a prevalent method for fabricating food packaging [28,29]. For instance, Ignacio Solaberrieta et al. developed electrospun nanofibers that can be utilized in active food packaging by incorporating *Aloe vera* skin extract (AVE) into poly (ethylene oxide) (PEO) [30]. When compared to traditional food packaging produced using solution casting technology, those generated through electrospun nanofibers offer distinct advantages, notably high porosity and a substantial specific surface area [31]. These properties facilitate the release of bioactive substances. It is worth noting that the spinnability of the solution and the morphology of the fibers are influenced by a multitude of electrospun parameters, including solution concentration, solvent volatility, conductivity, temperature, and humidity [32]. Konjac glucomannan (KGM), derived from konjac tubers, is a water-soluble polysaccharide [33]. It boasts commendable film-forming properties, biodegradability, biocompatibility, and safety [34]. However, performing individual electrostatic spinning with pure KGM is difficult [35]. To overcome this limitation, there is a need to identify a substance with superior spinnability. Fortunately, polyvinylpyrrolidone (PVP) is an amphoteric polymer with good spinnability and biocompatibility, providing a solid foundation for its effective blending with KGM [36]. For instance, Jaume Gomez. et al. combined PVA and PVP with natural mango kernel starch (MKS) in the fabrication of nanofibers to enhance its spinnability [37]. Moreover, Zhang Yin et al. fabricated electrospun compound nanofibers with PVP, PVA, and chitosan (CS). The evidence demonstrated that PVP could optimize the spinnability of the combination [38].

**Figure 1:**
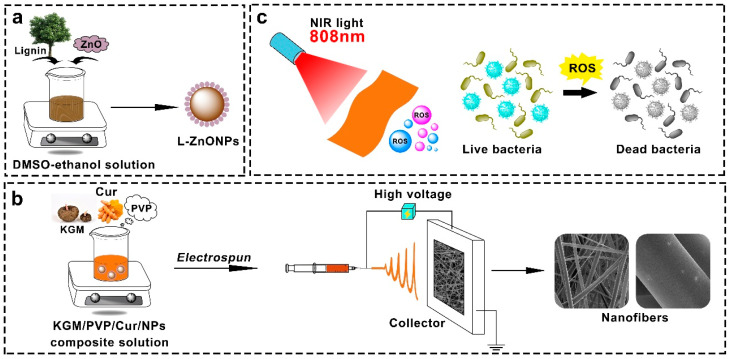
Schematic diagram of (**a**) synthesis of L-ZnONPs, (**b**) electrospun process, and (**c**) photodynamic antimicrobials.

While research on photodynamic antimicrobial activity is on the rise, most studies employ the solution casting technique for films preparation. In this study, we compounded lignin with Nano-ZnO to form L-ZnONPs. Subsequently, we utilized the electrospun technique to create KGM/PVP nanofibers loaded with Cur and L-ZnONPs, aimed at achieving photodynamic synergistic antibacterial activity (Figure 1). The morphology and structure of nanoparticles and nanofibers were characterized, and the thermal stability, mechanical properties, hydrophobicity, antioxidant properties, and photodynamic antimicrobial properties of nanofibers were investigated. Furthermore, the preservation effect of nanofibers in fresh strawberries was investigated. We believe that this research will promote the development of new food packaging approaches with highly efficient antibacterial activity for enhanced food preservation and safety.

## 2. Materials and Methods

### 2.1. Materials

Konjac glucomannan (KGM) powder (Purity ≥ 95%) was supplied from Yizhi Konjac Biological Technology Co., Ltd. (Wuhan, China). PVP (Mw = 1,300,000), Nano-zinc oxide (Nano-ZnO, Mw = 81.39), and lignin (Dealkaline) were acquired from Shanghai Aladdin Chemistry Co., Ltd. (Shanghai, China). Curcumin (Cur, Purity ≥ 98%, Mw = 368.38) was procured from Shanghai Maclean Biochemical Technology Co., Ltd. (Shanghai, China). Anhydrous ethanol, Span-80, Dimethyl sulfoxide (DMSO), calcium chloride (CaCl_2_) and Luria-Bertani (LB) agar were procured from Sinopharm Chemical Reagent Co., Ltd. (Shanghai, China).

### 2.2. Methods

#### 2.2.1. Synthesis of L-ZnONPs

The L-ZnONPs were prepared based on previous method with slight modifications [39]. Briefly, 0.5 g of lignin and Nano-ZnO were dissolved in 250 mL of DMSO and anhydrous ethanol, respectively. Then, they were mixed and magnetic stirred vigorously for 5 h. Subsequently, the L-ZnONPs were centrifuged, dried, and stored.

#### 2.2.2. Electrospun Solutions Preparation

Dissolved 1.0 g KGM powder in 80 mL ultrapure water, then added span-80 and 20 mL of ethanol solution drop by drop. Subsequently, the mixture was stirred in a water bath at 50 °C for 2 h. The PVP solution (10%) was stirred at ambient temperature overnight. The KGM/PVP sample was prepared by mixing PVP with KGM in a ratio of 9:1, stirring at 50 °C for 2 h and homogenizing. Afterwards, required amounts of Cur (Cur content: 1%) and L-ZnONPs (L-ZnONPs content: 1% and 2%) were added into above solutions to obtain electrospun solutions. All nanofibers were named KGM, KGM/PVP, KGM/PVP/2%L-ZnONPs, KGM/PVP/Cur/1%L-ZnONPs, KGM/PVP/Cur/2%L-ZnONPs, respectively.

#### 2.2.3. Electrospun Process

Nanofibers were prepared by using electrospun equipment. The electrospun specimens were injected into a 10 mL syringe with a needle (20-gauge), and the velocity was controlled at 0.2 mm/min, the spinning voltage was 9.0 kV, the needle-collector distance was 20 cm. Throughout the spinning process, the temperature and humidity remained constant at 55 °C and 25%, respectively.

#### 2.2.4. Characterization of L-ZnONPs

Fourier transform infrared spectroscopy (FTIR) was conducted to appraise the changes in the groups of the L-ZnONPs. The measurements were conducted using the KBr press method with 32 scans in the range of 4000–400 cm^−1^ at a resolution of 4 cm^−1^. Scanning electron microscopy (SEM) was performed to examine the micro morphology of the L-ZnONPs, with the samples coated with a thin layer of gold by sputtering prior to observation. Energy-dispersive X-ray spectroscopy (EDS) was analyzed mainly for the three elements C, Zn, and O.

#### 2.2.5. Characterization of Nanofibers

The morphology of the nanofibers was analyzed using SEM. The nanofibers were immobilized on an aluminum column and subjected to gold sputtering prior to the assay. The mean diameter and diameter distribution of the nanofibers were evaluated using the Image J, comprising 50 randomly selected fibers. EDS analyzes the elements C, Zn, and O in a similar format. The composition of nanofibers was measured by ATR-FTIR in the range (400–4000 cm^−1^) with 32 scans at a resolution of 4 cm^−1^. The crystalline structure of the nanofibers was measured by X-ray diffraction (XRD) in the range (10°–80°), with Cu Ka as the radiation source, operating at a voltage of 40 kV, 40 mA, and a scanning rate of 8°/min. Thermogravimetric analysis was conducted using thermo-gravimetric analysis (TGA) at a ramp velocity of 10 °C/min within the temperature range (30–800 °C) and an initial sample weight of approximately 5 mg. The recording of the initial temperature, residue, and maximum degradation temperature commenced at a sample weight loss of 1%. The tensile strength (TS) and elongation at break (EAB) were tested using a tensile tester (Kyoto, Japan). Prior to testing, the nanofibers were slit into small rectangular specimens measuring 1 × 5 cm, and the thickness was measured at five random locations using an electronic micrometer (DITRON, Chengdu, China). The water contact angle (WCA) of the nanofibers was recorded at room temperature using a WCA analyzer. A drop (10 µL) of ultrapure water was placed on the nanofibers and photographed within 5 s.

Water vapor permeability (WVP) measurements were conducted following the previously described method with minor modifications [40]. The nanofibers were loaded on top of a weighing bottle holding anhydrous CaCl_2_ (3.0 g), and the bottle was maintained in a room temperature, 70% RH container and each 24 h was weighed and computed as follows:

(1) $$\mathrm{WVP}=\frac{\Delta W\times x}{A\times t\times \Delta P}$$

where ΔW (g) means the total weight discrepancy between the weighing bottle and the sample, x (mm) means the average thickness, A (mm^2^) means the experimental space, t (24 h) means the experimental time, and ΔP (Pa) means the vapor pressure discrepancy between the nanofibers and the environment.

#### 2.2.6. Antioxidant Performance Evaluation (ABTS Scavenging)

The antioxidant capacity of nanofibers was determined by ABTS free radicals scavenging rate [24]. The nanofibers (50 mg) were dispersed in an anhydrous ethanol solution (5 mL). After that, it was shaken for 1 h. The leachate of nanofibers was admixed with ABTS solution in a ratio (4:0.2) and reactive for 5 min in a dark environment. Finally, the absorption of the reaction liquids was recorded with a UV spectrophotometer (UV-2600, Kyoto, Japan) at 734 nm. The rate of ABTS free radical scavenging was computed as shown below:

(2) $$\mathrm{ABTS}\;\mathrm{free}\;\mathrm{radical}\;\mathrm{scavenging}\;\mathrm{rate}=\frac{A_{0}-A_{1}}{A_{0}}$$

where A_0_ was the absorption of the ABTS solution and A_1_ was the absorption of the reaction liquids.

#### 2.2.7. Photodynamic Antimicrobial Properties

The antimicrobial properties of nanofibers were determined by agar diffusion method. Briefly, the nanofibers were chopped into slices (diameter = 6.0 mm) and placed on agar medium that had been inoculated with *S. aureus* and *E. coli* (inoculum concentration: 10^5^ CFU/mL). Then, the nanofibers were irradiated with red light of 808 nm for 30 min. Finally, they were incubated at 37 °C for 16 h.

#### 2.2.8. Application of Nanofibers in Strawberry Preservation

This study aimed to estimate the efficacy of nanofibers in maintaining the freshness of strawberries. The experimental groups consisted of strawberries wrapped in KGM, KGM/PVP, KGM/PVP/2%L-ZnONPs, KGM/PVP/Cur/1%L-ZnONPs, and KGM/PVP/Cur/2%L-ZnONPs nanofibers, while the control group was left untreated. The strawberries were stored at room temperature, and their appearance was photographed at 1, 3, 5, and 7 days to observe any changes. Additionally, the strawberries were evaluated for weight loss, hardness loss, and pH before and after storage for 7 days.

### 2.3. Statistical Analysis

A minimum of three independent experiments were conducted for each experiment. All experimental data analyses were conducted using Origin 2021 (version 2021, Northampton, MA, USA).

## 3. Results and Discussion

### 3.1. Characterization of L-ZnONPs

**Figure 2:**
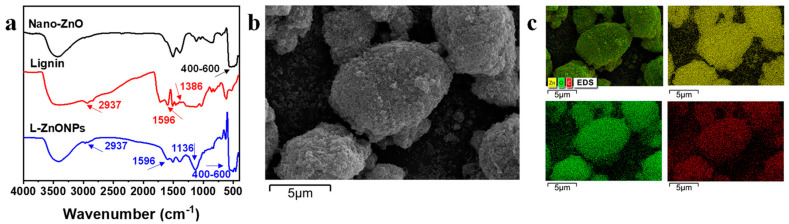
(**a**) FTIR spectra of the Nano-ZnO, Lignin, and L-ZnONPs. (**b**) SEM image of the L-ZnONPs. (**c**) EDS image of the L-ZnONPs.

The conformations and changes in the functional groups of the nanoparticles are studied by FTIR spectra. In Figure 2a, the FTIR spectral peaks of Nano-ZnO display prominent broad peaks (400–600 cm^−1^), which correspond to the Zn-O stretching vibrations in Nano-ZnO [41]. Additionally, a peak at 3427 cm^−1^ is indicative of O-H stretching. In the spectra of lignin, characteristic peaks are located at 3400 cm^−1^ corresponding to -OH stretching, 2937 cm^−1^ is attributed to -CH_2_ stretching, 1386 cm^−1^ and 1596 cm^−1^ are interpreted as -C=O stretching and phenyl ring backbone absorption peaks [42]. In the spectrum of L-ZnONPs, the distinctive peaks of both ZnO and lignin are discernible. Notably, strong vibrational peaks appearing at 1136 cm^−1^ suggest the strong interaction occurring between lignin and Nano-ZnO. These findings support the successful integration of lignin and Nano-ZnO.

Figure 2b,c show the SEM images and EDS images of L-ZnONPs, respectively. The SEM images depict the ellipsoidal architecture of the nanoparticles, featuring both large and small particles. Furthermore, the surface of the nanoparticles is rough and porous; these characteristics contribute to an enlarged specific surface area. Moreover, Nano-ZnO appears to be firmly adhered to the lignin surface, consistent with previous research [43]. The EDS mapping images of the L-ZnONPs illustrate the partitioning of C, Zn, and O. Notably, these three elements (C, Zn, and O) exhibit a uniform distribution, providing further evidence of the successful combination of lignin and Nano-ZnO.

### 3.2. Morphological Analysis of Nanofibers

**Figure 3:**
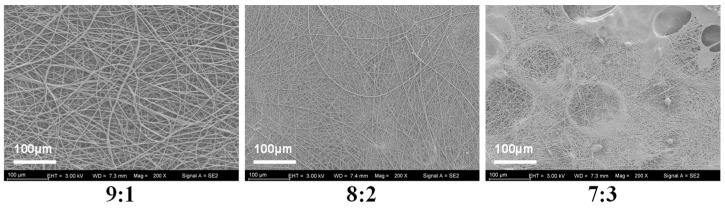
SEM images of nanofibers with different mixing ratios (PVP:KGM).

To determine the optimal mixing ratio of PVP and KGM solutions, we spun the nanofibers in three ratios of 9:1, 8:2, and 7:3 and evaluated the spinning effect by examining the microscopic morphology of the nanofibers (Figure 3). It shows that nanofibers spun in a ratio of 8:2 exhibit tangles and droplets. Additionally, nanofibers spun with a 7:3 ratio show even more droplets and tangles. Fortunately, the nanofibers spun in the 9:1 ratio have a good shape and are without liquid droplets or tangles. Therefore, the 9:1 ratio was chosen for subsequent experiments.

**Figure 4:**
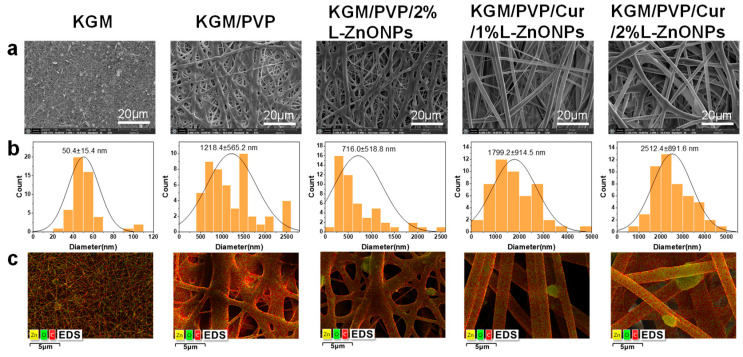
(**a**) SEM images of nanofibers. (**b**) SEM images corresponding to the histogram of the diameter distribution. (**c**) Elemental mapping image of nanofibers.

Figure 4a–c showcases SEM images, diameter distribution histograms, and EDS mapping images of nanofibers. Pure KGM nanofibers exhibit a significant presence of beaded and fractured fibers. This can be accounted for by the inadequate evaporation of the solvent during the electrospun process and the limited spinnability of KGM itself. In contrast, both KGM/PVP and KGM/PVP/2%L-ZnONPs nanofibers display no tangles or droplets but rough surfaces. This phenomenon may be the result of the fiber structure collapsing due to rapid moisture absorption [44]. In addition, the absence of beading and fiber fractures can be traced to the reduction in interactions between KGM molecules facilitated by PVP, the phenomenon supported by prior research [45]. However, upon the addition of Cur, both KGM/PVP/Cur/1%L-ZnONPs and KGM/PVP/Cur/2%L-ZnONPs nanofibers exhibit smooth surfaces. This change in nanofiber morphology may be ascribed to the hydrophobic nature of Cur, which enhances the fiber structure.

The average diameters of the various types of nanofibers are as follows: KGM nanofibers, KGM/PVP nanofibers, KGM/PVP/2%L-ZnONPs nanofibers, KGM/PVP/Cur/1%L-ZnONPs nanofibers, and KGM/PVP/Cur/2%L-ZnONPs nanofibers are 50.4 ± 15.4 nm, 1218.4 ± 565.2 nm, 716.0 ± 518.8 nm, 1799.2 ± 914.5 nm, and 2512.4 ± 891.6 nm, respectively. Herein, the alteration in nanofibers diameter may be attributed to a change in the viscosity of the electrospun solutions [46].

The EDS figures show the average representation of the distribution of the three elements (C, Zn, and O) within the nanofibers. The images clearly demonstrate that the nanoparticles are distributed both within and on the surface of the nanofibers. Furthermore, the apparent white bright spots observed on the surface of the nanofibers were confirmed to correspond to L-ZnONPs.

### 3.3. Group Changes Analysis

**Figure 5:**
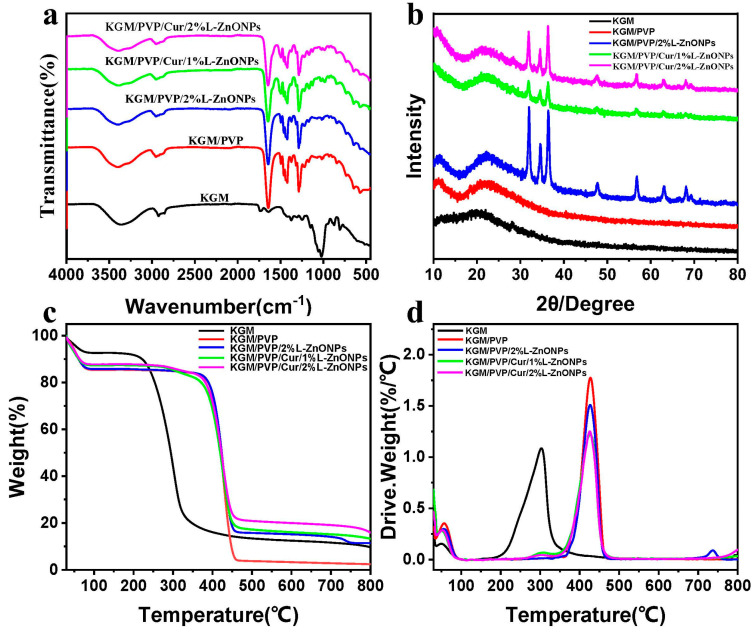
(**a**) ATR-FTIR spectra of nanofibers. (**b**) XRD spectra of nanofibers. (**c**,**d**) TGA and DTG curves of nanofibers.

In Figure 5a, the ATR-FTIR spectrum of the nanofibers is presented. For pure KGM nanofibers, the eigenpeak at 3359 cm^−1^ corresponds to the stretch vibration of -OH, while the eigenpeaks at 2930 cm^−1^ and 1027 cm^−1^ are associated with the stretching vibration of C-H and C-O, separately [47,48]. Additionally, the eigenpeak at 1640 cm^−1^ is linked to the stretching vibration of intramolecular hydrogen bonding, while the eigenpeak at 1730 cm^−1^ relates to the vibrational stretching of the C=O within the KGM acetyl group and the C-O group within intermolecular hydrogen bonding [48,49]. Furthermore, the eigenpeaks at 874 cm^−1^ and 807 cm^−1^ are connected to the vibrational stretching of the mannan unit of the KGM [45,50]. In the case of KGM/PVP nanofibers, characteristic peaks of PVP are observed in addition to those of KGM. Specifically, the peak at 1495 cm^−1^ is assigned to the vibrational stretching of C-N and the bending vibration of N-H, while the peaks at 1424 and 1291 cm^−1^ are linked to the bending vibration of C-H and C-N, correspondingly [51,52]. In addition, the eigenpeaks shift from 3359 to 3397 cm^−1^ and from 1640 to 1642 cm^−1^ with changes in transmittance, indicating an enhancement of molecular interaction between KGM and PVP through hydrogen bonding [53]. For KGM/PVP/2%L-ZnONPs, no new functional groups are observed apart from the characteristic peaks of KGM, PVP, and nanoparticles, which suggests that there are solely physical interactions between the nanoparticles and the KGM/PVP nanofibers matrix. However, the characteristic peaks become less pronounced in the nanofibers with the addition of Cur, which may be due to the encapsulation of Cur within the structure formed by the nanofiber matrix, restricting the stretching vibrations of the functional groups [24].

### 3.4. XRD Analysis

In Figure 5b, the diffraction patterns of the different nanofiber samples are depicted. The KGM nanofibers show a broad diffraction peak at 20.3°, indicating the amorphous nature KGM [45]. In the case of KGM/PVP, a broad diffraction peak at 22° is observed, slightly shifted compared to KGM. This shift is attributed to the interaction of KGM with PVP, which is consistent with the ATR-FTIR results [54]. In addition, the diffraction pattern of KGM/PVP nanofibers closely resembled that of pure KGM, indicating good compatibility between KGM and PVP. Upon the addition of nanoparticles, the nanofibers showed crystallization peaks at 31.98°, 34.66°, 36.42°, 47.78°, 56.88°, 63.16°, and 68.12°. These peaks align well with the diffraction patterns of ZnO in the nanoparticles, corresponding to (100), (002), (101), (102), (110), (103), and (112) crystallographic planes (JCPDS card: No. 36-1451) [20].

### 3.5. Thermal Performance Analysis

Figure 5c,d show the TGA and DTG curves of the nanofibers, respectively, providing insights into their thermal stability. The thermal decomposition of these nanofibers can be categorized into three main steps. The initial step (<100 °C) which involves weight loss is attributable to the removal of unbound water [55]. The second stage (200–320 °C) of thermal decomposition in KGM nanofibers is associated with the elimination of hydrogen bonds and the decomposition of sugar rings in KGM [50]. However, in the case of KGM/PVP nanofibers, this second decomposition stage extends up to 460 °C, owing to the establishment of hydrogen bonds between KGM and PVP, which enhance the thermal stability of the nanofibers [48]. This observation aligns with the results from ATR-FTIR analysis. The weight loss in the third stage remains elative constant but slightly decreases, indicating further decomposition of molecular weight [44].

### 3.6. Mechanical Performance Analysis

**Figure 6:**
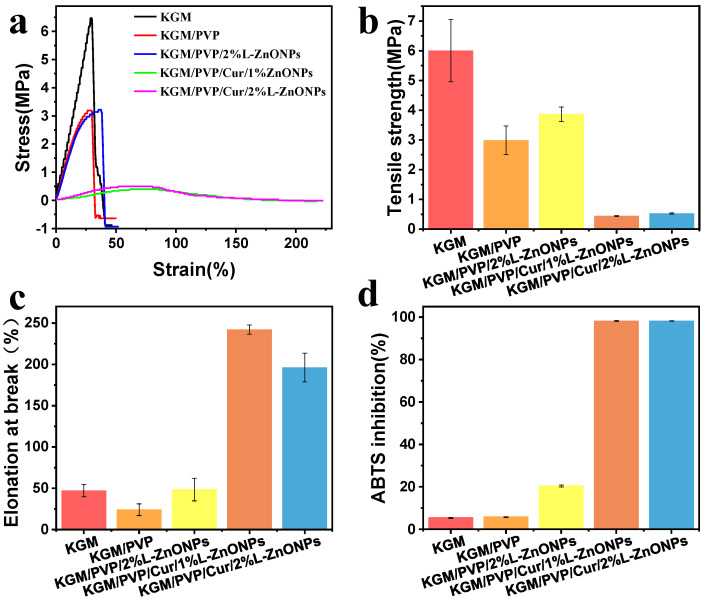
Mechanical properties of nanofibers: (**a**) stress–strain versus strain graphs, (**b**) TS, (**c**) EAB. (**d**) The antioxidant activity of nanofibers based on ABTS method.

The stress–strain versus strain graph, TS, and EAB of the nanofibers are depicted in Figure 6a–c. The TS of KGM/PVP nanofibers is inferior to that of pure KGM. This may be attributed to the finer average diameter and higher packing density of pure KGM nanofibers [56]. Normally, enhancing both TS and EAB of composites simultaneously can be challenging [53,57]. However, it is interesting to note that doping the nanofibers with nanoparticles leads to improvements in both TS and EAB. This enhancement can be owed to the homogeneous distribution of nanoparticles in the nanofibers [58]. A similar effect was identified by Wang et al., who found that QLS/ZnO NCs could upgrade the mechanical performance of PU films [59]. Conversely, the incorporation of Cur resulted in a deterioration of the TS of the nanofibers. However, it significantly enhanced the EAB (>241%), indicating that the nanofibers exhibited good tensile properties. These results suggest that KGM/PVP/Cur/L-ZnONPs nanofibers possess good ductility but lower strength.

### 3.7. Antioxidant Activities Analysis

The antioxidant potential of the nanofibers was assessed using ABTS free radical scavenging assay, and the results are illustrated in Figure 6d. The ABTS radical scavenging rates for the KGM and KGM/PVP nanofibers are only 5.48% and 5.87%, respectively. However, upon the incorporation of nanoparticles, the ABTS radical scavenging rate increased to 20.48%. This increase can be accredited to the intervention of phenolic hydroxyl groups in the lignin within the nanoparticles [60]. Furthermore, the addition of Cur resulted in a remarkable enhancement of the antioxidant competence of the nanofibers, with ABTS radical scavenging rates of 98.14% and 98.19%, respectively. This is associated with the presence of an abundance of phenolic hydroxyl groups within the Cur molecule, as the essence of free radical scavenging involves the transfer of phenolic hydroxyl hydrogen atoms [61]. The results indicate that the prepared nanofibers demonstrate excellent antioxidant capacity and have potential applications for inhibiting oxidative spoilage.

### 3.8. Water Contact Angle (WCA) Analysis

**Figure 7:**
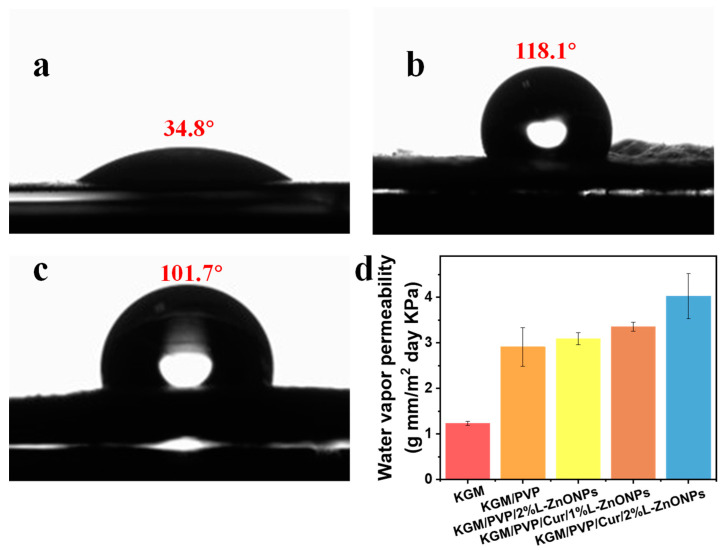
WCA of (**a**) KGM, (**b**) KGM/PVP/Cur/1%L-ZnONPs, and (**c**) KGM/PVP/Cur/2%L-ZnONPs nanofibers. (**d**) WVP of nanofibers.

Surface wettability is a crucial parameter for food packaging, and hydrophobic food packaging is more suitable for practical applications [62]. The WCA measurements for the pure KGM nanofibers, KGM/PVP/Cur/1% L-ZnONPs nanofibers, and KGM/PVP/Cur/2% L-ZnONPs nanofibers are 34.8°, 118.1°, and 101.7°, respectively (Figure 7a–c). The increase in WCA of the nanofibers after adding Cur can be attributed to the fact that Cur is a naturally hydrophobic polyphenol [63]. The hydrophobic benzene ring within Cur has a more pronounced effect on the nanofibers than the polar hydroxyl group [64]. Conversely, the incorporation of nanoparticles induced the decrease in the WCA of the nanofibers, which can be attributed to the surface hydrophilicity of the nanoparticles. This phenomenon has also been demonstrated by Daniele Del Buono et al. [65]. Nanofibers are considered hydrophobic materials if they have a WCA value of ≥90° [46]. Hydrophobic materials possess excellent water repellent properties, which are essential for preventing moisture loss and are critical in food packaging [66].

### 3.9. WVP Assessments Analysis

WVP directly impacts the interplay between food and the storage environment. Its magnitude is influenced not only by the chemical structure of the nanofibers but also by the hydrophilic–hydrophobic characteristics of these nanofibers [34,50]. Figure 7d shows the WVP of nanofibers. The WVP value of pure KGM nanofibers was measured at 1.23 ± 0.04 (g mm/m^2^ day KPa), while the WVP value of KGM/PVP nanofibers increased to 2.91 ± 0.42 (g mm/m^2^ day KPa), which can be explained as a result of the generation of hydrogen bonds [46]. Additionally, the high hydrophilicity of PVP may increase the WVP of the nanofibers. The WVP of the nanofibers also remained elevated after the addition of L-ZnONPs, which can be attributed to the hydrophilic hydroxyl moieties of Nano-ZnO, increasing the hydrophilicity of the nanofibers and the moisture vapor sorption sites [67]. The WVP of KGM/PVP/Cur/L-ZnONPs was found to be acceptable in this study. Our nanofibers have WVP values similar to polypropylene (PP) films (3.9–6.2 g mm/m^2^ day KPa), much lower than polystyrene films (109–155 g mm/m^2^ day KPa), but also higher than PVC (0.94–0.95 g mm/m^2^ day KPa) and PLA (1.34 g mm/m^2^ day KPa) prepared films [68].

### 3.10. Photodynamic Antimicrobial Activity Analysis

**Figure 8:**
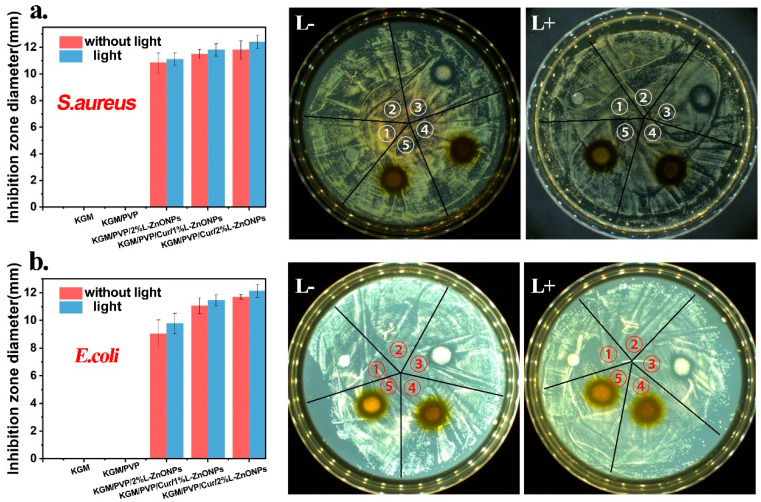
The antibacterial activity of nanofibers against (**a**) *S. aureus* and (**b**) *E. coli* (➀ KGM, ➁ KGM/PVP, ➂ KGM/PVP/2%L-ZnONPs, ➃ KGM/PVP/Cur/1%L-ZnONPs, ➄ KGM/PVP/Cur/2%L-ZnONPs).

The photodynamic antimicrobial activity of nanofibers against *S. aureus* and *E. coli* is investigated and the outcomes are presented in Figure 8a,b. Typically, a larger inhibition zone around the nanofiber discs indicates a more effective antibacterial activity of the nanofibers [57]. It is observed that there is no inhibition zone around the KGM and KGM/PVP nanofibers discs, indicating that they lacked antimicrobial activity. However, the nanofibers containing nanoparticles and Cur displayed substantial antimicrobial capabilities against both *S. aureus* and *E. coli* bacteria. Furthermore, after irradiation with 808 nm red light, the inhibition zone around the nanofibers is larger, indicating that irradiation could enhance the antibacterial activity of the nanofibers. This enhancement may be due to the inhibitory effect on bacterial growth resulting from the presence of nanoparticles and Cur in the nanofibers after light irradiation, leading to the production of ROS [59].

### 3.11. Preservation Effects for Strawberries Analysis

**Figure 9:**
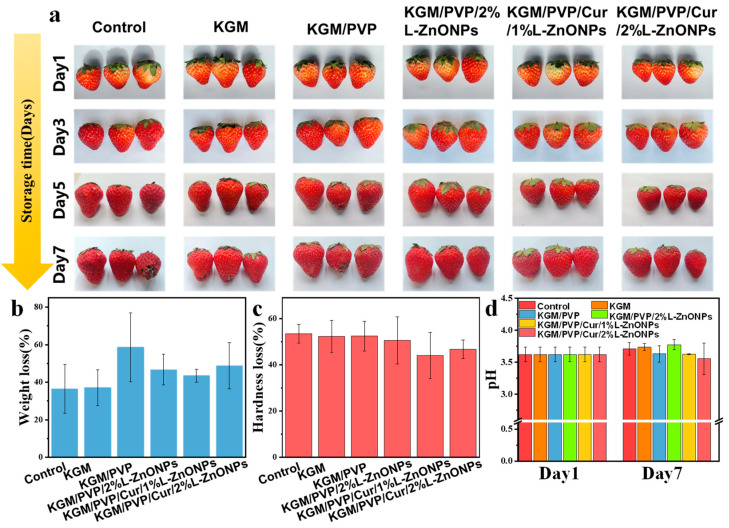
(**a**) The changes in visual appearance; (**b**) weight loss; (**c**) hardness loss; and (**d**) pH changes of strawberries before and after storage for 7 days.

We assessed the impact of nanofibers wrapping on the quality of fresh fruits. Figure 9 shows the preservation effect of nanofibers on fresh strawberries. Strawberries wrapped in control, KGM, and KGM/PVP films exhibited slight decay and deterioration, while those wrapped in KGM/PVP/2%L-ZnONPs, KGM/PVP/Cur/1%L-ZnONPs, and KGM/PVP/Cur/2%L-ZnONPs nanofibers maintained good appearance. After 7 days of storage, all samples experienced a loss of quality due to moisture loss and nutrient depletion of the strawberries [69]. Furthermore, we discovered that treating the strawberries with films of KGM/PVP/Cur/1%L-ZnONPs and KGM/PVP/Cur/2%L-ZnONPs significantly reduced the rate of hardness loss. The pH of the strawberries increased at the end of storage due to the depletion of organic acids during metabolism and respiration [70]. However, the pH of strawberries wrapped in KGM/PVP/Cur/1%L-ZnONPs and KGM/PVP/Cur/2%L-ZnONPs nanofibers was significantly inferior to that of the other groups. This is on account of the inhibition of strawberry respiration caused by the sustained release of L-ZnONPs and Cur. Therefore, we have concluded that the nanofibers have a certain preservation effect on the strawberries. Although the weight loss of strawberries slightly increased after treatment with the films, the nanofibers were able to prevent the growth of microorganisms, lessen the loss of strawberry hardness, and inhibit the consumption of organic acids.

## 4. Conclusions

In this work, we successfully prepared KGM/PVP/Cur/L-ZnONPs nanofibers with potent photodynamic antimicrobial properties using the electrospun technique. These nanofibers exhibited significant enhancements in the thermal stability and hydrophobicity. Moreover, their improved flexibility indicated robust resistance to deformation. Notably, under 808 nm red light irradiation, the antimicrobial efficacy of nanofibers was improved, leading to enhanced inhibition of both *S. aureus* (inhibition: 12.4 mm) and *E. coli* (12.1 mm). This renders food less susceptible to contamination by *S. aureus* and *E. coli*. Additionally, the nanofibers have a certain freshness-preserving effect on strawberries, inhibiting the onset of rotting and spoilage, thereby extending the shelf life. This work a fortiori holds several advantages, including cost-effectiveness and straightforward preparation, which lays a solid foundation for the development of novel food packaging with highly efficient antimicrobial properties.

## Data Availability

The data presented in this study are available on request from the corresponding author. The data are not publicly available due to privacy restrictions.
